# FAIR enough: Building an academic data ecosystem to make real-world data available for translational research

**DOI:** 10.1017/cts.2024.530

**Published:** 2024-05-02

**Authors:** Isabella Chu, Rebecca Miller, Ian Mathews, Ayin Vala, Lesley Sept, Ruth O’Hara, David H. Rehkopf

**Affiliations:** 1 The Stanford Center for Population Health Sciences, Stanford School of Medicine, Stanford University, Palo Alto, CA, USA; 2 Redivis, Inc, Oakland, CA, USA; 3 Psychiatry and Behavioral Sciences, Stanford School of Medicine, Stanford University, Stanford, CA, USA; 4 Veterans Administration Palo Alto Health Care System, Sierra Pacific Mental Illness, Research, Education, and Clinical Center, Palo Alto, CA, USA; 5 Department of Epidemiology and Population Health, Stanford School of Medicine, Stanford University, Stanford, CA, USA; 6 Department of Medicine, Division of Primary Care and Population Health, Stanford School of Medicine, Stanford University, Stanford, CA, USA; 7 Department of Pediatrics, Stanford School of Medicine, Stanford University, Stanford, CA, USA; 8 Department of Health Policy, Stanford School of Medicine, Stanford University, Stanford, CA, USA; 9 Department of Sociology, Stanford University, Stanford, CA, USA

**Keywords:** Clinical and translational science awards (CTSA), claims, data management, electronic health records (EHR), real-world data, translational research

## Abstract

The Stanford Population Health Sciences Data Ecosystem was created to facilitate the use of large datasets containing health records from hundreds of millions of individuals. This necessitated technical solutions optimized for an academic medical center to manage and share high-risk data at scale. Through collaboration with internal and external partners, we have built a Data Ecosystem to host, curate, and share data with hundreds of users in a secure and compliant manner. This platform has enabled us to host unique data assets and serve the needs of researchers across Stanford University, and the technology and approach were designed to be replicable and portable to other institutions. We have found, however, that though these technological advances are necessary, they are not sufficient. Challenges around making data Findable, Accessible, Interoperable, and Reusable remain. Our experience has demonstrated that there is a high demand for access to real-world data, and that if the appropriate tools and structures are in place, translational research can be advanced considerably. Together, technological solutions, management structures, and education to support researcher, data science, and community collaborations offer more impactful processes over the long-term for supporting translational research with real-world data.

The Stanford Center for Population Health Sciences (S-PHS) was founded in 2015, with the mission to “improve the health of populations by bringing together diverse disciplines and data to understand and address social, environmental, behavioral, and biological factors on both a domestic and global scale [1].” One of the central means to achieve that mission was to advance translational science by providing access to real-world data (RWD). We sought to create structures and processes that would promote impactful translational science using RWD, while acknowledging all the complexities of the data, and the new knowledge and skills needed to use RWD effectively. In this article, we first briefly describe some of the primary benefits of RWD that motivated this effort, along with the major challenges of using RWD for translational research. We then describe the process of addressing the challenges through developing a data ecosystem for utilizing RWD in the academic setting. We set out to solve these problems not just for our own organization, but also for others by creating a solution that would be replicable and affordable for large research groups, and other academic and nonprofit organizations seeking to utilize high-value, high-risk data in a secure, scalable, and compliant manner. Thirdly, we discuss new directions of interest based on lessons learned, and what we see as critical next steps for increasing the impact of RWD to solve pressing population health and health equity problems. We share what we have learned over the past eight years about how best to facilitate the use of these data for translational science, and what we think still needs to happen to maximize RWD impact for improving population health and reducing health inequities. We hope this past pathway and our observations on looking forward will be of interest to research groups and institutions that are interested in utilizing RWD to advance translational science.

## Rationale for a focus on real-world data

Real-world data can be defined most generally as data that was collected for a purpose other than for research. As this data relates to health, this most notably includes data from wearable devices, billing data, and data from electronic health records (EHR). These data have several advantages over the data used in traditional research studies. Many of these advantages are because these data typically include a much larger number of individuals than is possible to collect when done for a study designed specifically for research purposes. Thus, there is greater statistical power in RWD to identify small but important impacts of exposures and treatments, both advantageous and adverse. RWD are better able to support a new generation of neural network models, like transformer models, that fit massive numbers of nodes to data and require large sample sizes to produce meaningful findings. Real-world data with large numbers of individuals are also much better for precision health approaches to treatment [2], given their increased ability to have stable findings within subgroups of the population. Relatedly, they tend to allow for more quantitative intersectional approaches to studying health because there are enough individuals in subgroups of the population to estimate the heterogeneity of treatment effects [3]. Prospectively collecting data from an equivalent number of individuals for a research project would be prohibitively expensive, whereas the costs of collecting RWD are typically already paid for by the group that used the data for its intended non-research purpose. A further advantage of RWD is the multipurpose nature of types of data that make the study of a wide range of health topics feasible. When permitted, linkage with other datasets can enable tracking of patients across many years, something that can be done instantaneously and retrospectively, without waiting decades (as is the case with the traditional prospective, longitudinal study design structures). Many RWD sets, although not all, contain more diverse populations that are closer to being representative of target populations since enrollment and inclusion in the data are often the default. Finally, RWD typically is continuously and constantly being updated, allowing more up-to-date insights into health risks and protective factors. This also makes RWD critical for adapting and improving patient care in real time, as it is paramount to understanding health systems.

## Challenges of using real-world data

Three major challenges of using RWD are: (1) the computational difficulties that come with the large size of the data, (2) the need to keep proprietary data collected for another use private and secure, and (3) the fact that RWD have very different forms of bias and measurement error than data that is intentionally collected for research purposes. At many terabytes each, RWD typically exceed the capacities of most personal computers. Like almost all health-related data, RWD has a risk of re-identification of individuals, so the data must be kept secure. What is unique about RWD, however, is that there are often also proprietary risks of disclosure because the data were not collected by researchers themselves, but by another entity—often a private entity. Many datasets have specific stipulations on who may use the data and how, such as limiting what research questions may be asked, or what affiliations or roles researchers must have (e.g. requiring affiliation with the licensing organization, or prohibiting the use of data for trainee projects). The selection and measurement errors in RWD have been well described, most notably finding that with claims data and EHR data, patients will typically only be observed when they are having health problems; consequently, identifying the population that is at risk is often a challenge in open cohorts, and clinical definitions of health outcomes are often not included, since that is not the purpose of many types of RWD[4].

## Our approaches to addressing real-world data access and computation challenges

To effectively meet the needs of researchers using RWD, we focused on bringing together the following:

- Data whose data use agreements can allow a large number of people to use and reuse the data for a wide range of projects;
- Data administration and governance that is transparent, tractable, and protects the privacy and confidentiality of individuals in datasets, and also constrains researchers from violating policies, regulations, or contractual obligations;
- Data management and expertise to ensure data are curated in a way that is practical for research;
- Computational environments and software that are secure, powerful, flexible, and scalable.

Wilkinson et al. set a series of standards to ensure maximum utility of data – namely that they must be Findable, Accessible, Interoperable, and Reusable (FAIR) [5]. We have reinterpreted these FAIR standards for high-risk data as follows:

- Findable: Entities should issue a unique digital object identifier (DOI) for each iteration of a dataset. The data should be annotated, tagged, and indexed in such a way that they are optimized for commonly used search engines. Specifically, metadata should be presented in a machine-readable format utilizing standard schemata and vocabularies.
- Accessible: There should be a transparent, standardized process for obtaining data access and using data that is closely monitored and managed by a data custodian that meets all relevant regulatory and security requirements, including those specific to individual datasets and/or high-risk datasets. Metadata must be retrievable by their identifier using standardized communications, and sufficient to enable prospective users to evaluate the data’s utility for their research question(s), particularly if the data have a high barrier to access.
- Interoperable: Data and metadata use a formal, accessible, shared, and broadly applicable language for knowledge representation that follows FAIR principles, and include qualified references to other data.
- Reusable: Data and metadata should be well described, released with clear parameters around provenance and licensure, and meet domain-relevant standards.

**Figure 1.**
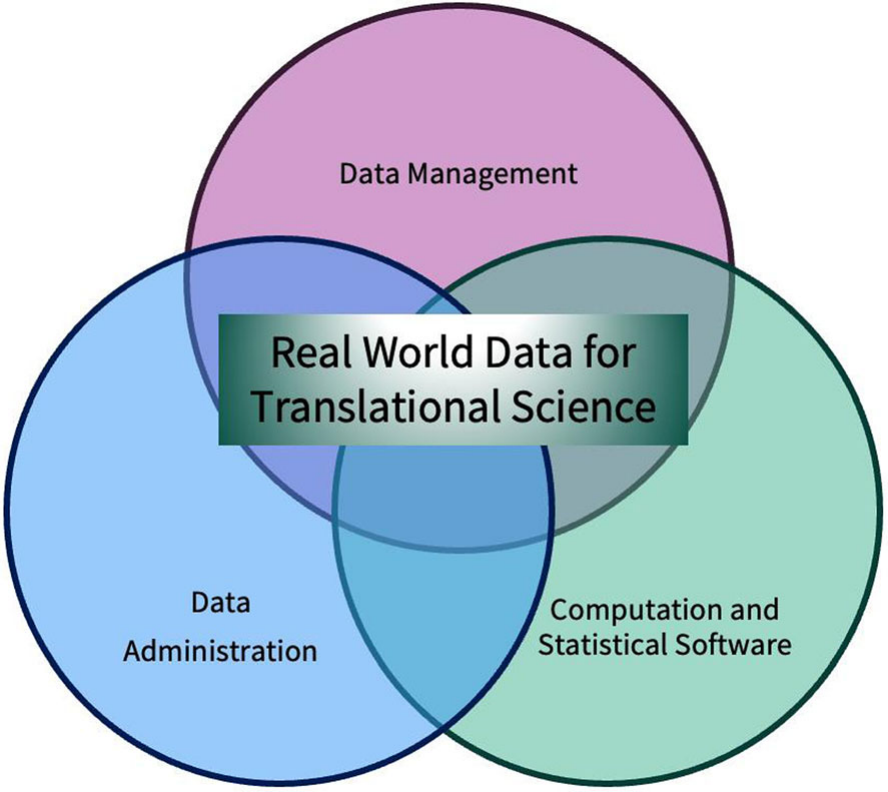
Three pillars of making real-world data useful.

At the time of S-PHS’s inception, there were no products tailored or affordable to academic markets for achieving these objectives. S-PHS also needed to manage complex data access requirements, and maintain visibility and direct control of S-PHS-hosted data. S-PHS worked with a startup, Redivis [6], to build a platform that could handle data discovery, access, curation, annotation, sharing, exploration, and visualization. In parallel, the Stanford Research Computing Center built a secure, customizable, and scalable computational environment based on the Google Cloud Platform. Data curation, annotation, and administration were handled by S-PHS staff. Together, these three pillars of data management form the core essentials (Fig. 1) of what is needed for clinical researchers, epidemiologists, and statisticians who do not have advanced large database data science skills to use RWD.

In the time between S-PHS’s initial investment and now, other platforms have emerged that provide some similar features. Many of these have been funded or are directly operated by the NIH. Examples include Vivli, N3C, and All of Us [7–9]. A team from Harvard built Aetion, which specializes in models for clinical research [10,11]. These systems share several features in common with the S-PHS system. Scalability of these systems is achieved with commercial cloud. In almost all cases, partnership with private entities was also used to build each system, and in many cases, for ongoing operation. For example, the All of Us Data and Research Center was achieved through a collaboration with Verily Life Sciences [12], and the National COVID Cohort Collaborative (N3C) collaborated with Palantir [13]. In the private sector, platforms such as Snowflake and TriNetX also make data available, and owners of claims data such as IQVIA, Optum, and MarketScan use proprietary, in-house platforms. Similarly to Redivis, these platforms offer a secure, scalable, and customizable computational environment with tiered access depending on the researchers' expertise and needs. Many of these platforms have lowered barriers to access by making only de-identified data generally available to investigators, though Research Identifiable Files may be available to smaller groups of researchers [12].

Perhaps the most important distinction of our data platform is that it was optimized for academic data centers and designed to be replicable: an affordable, off-the-shelf software [14] — and indeed, Redivis has been adopted by Stanford University Libraries, and several other university libraries and data centers. A feature particularly important to academic use is that it is implemented as a multi-tenant environment, which, in practice, means that researchers can discover and combine any dataset that they have access to across institutions that are utilizing this software (if the data owners have approved such linkages). This has the potential to encourage sharing of high-risk data since the data custodian can share the data without having to relinquish branding, credit, or control.

Redivis is unique in its particularly robust permissioning system and the level of complexity that it supports. Most labs work with multiple datasets with varying risk levels and access requirements. For example, a lab with eight researchers may have a study with four high-risk datasets — two of which require proof of project approval from the data owners, three of which require IRB approval, and all of which require proof of encryption — to access. The Redivis system can manage this complexity, and allow team members to share projects, data, and code in the project tool. It automatically restricts access to each dataset based on the tier (view, metadata, 1% sample, or full dataset) for which the team member is authorized. This enables us to remain compliant without having to homogenize the risk profiles or access requirements of datasets within a project, and allows teams to work together in an organic way.

## Incorporating FAIR principles into our technical infrastructure

### Findable

**Figure 2.**
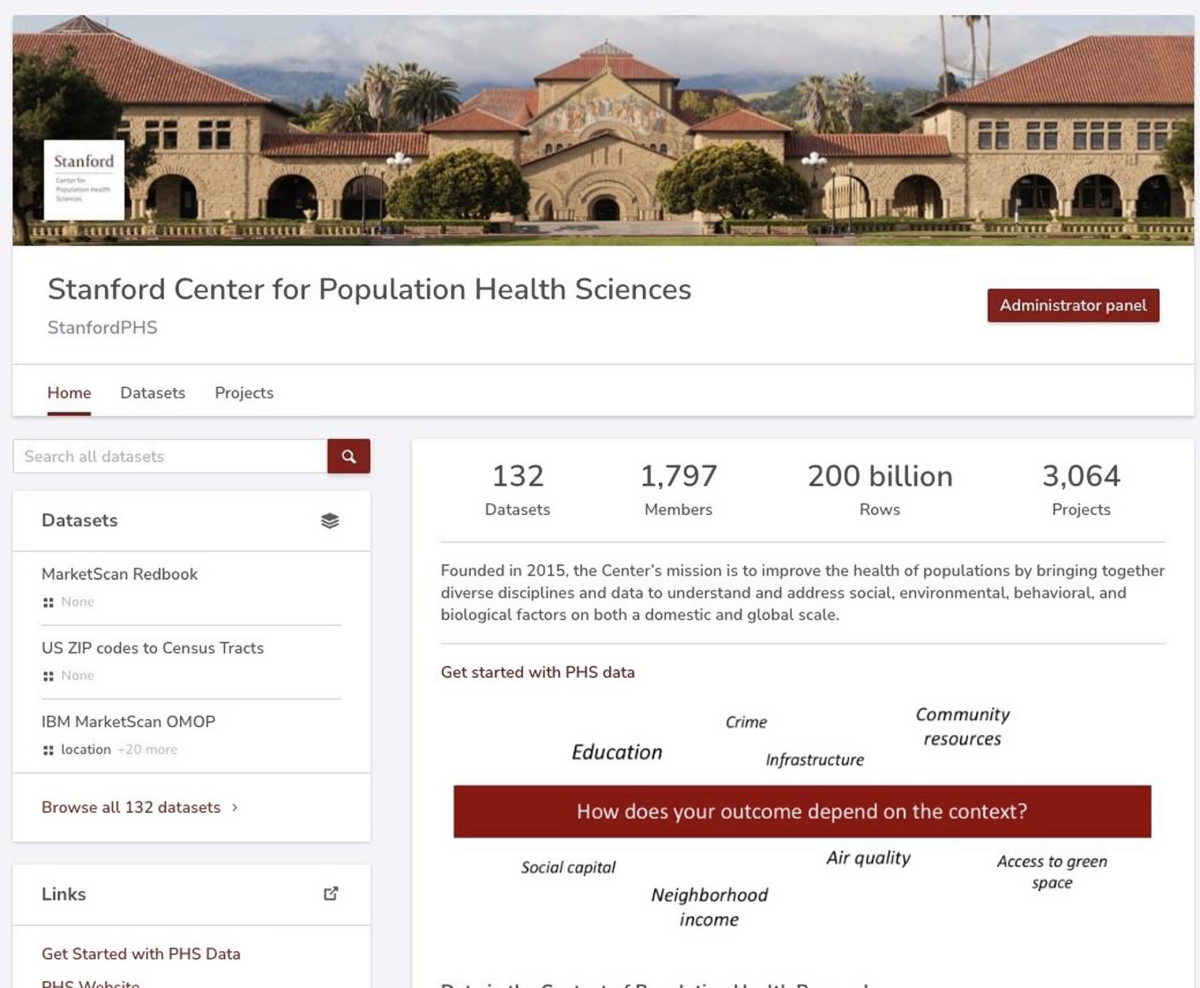
Landing page of the S-PHS Data Portal.

The entry point to data is the S-PHS Data Portal (Fig. 2), which enables data search within the platform using either natural language or common ontologies, manages access as described above, and enables investigators to explore data. In addition to managing data discovery and access, it has tools that enable researchers to explore a wide array of dataset types across a federated system of data lakes and enclaves. It also indexes all documentation, tags, variables, and variable metadata across each dataset, allowing the system to return the dataset(s) relevant to a researcher’s line of inquiry. For example, if a medical diagnosis variable in a dataset is annotated as containing International Classification of Disease, Tenth Revision (ICD-X) diagnosis codes, and includes observations in that variable of the value “I26” (pulmonary embolism), any searches for “pulmonary” will return this dataset, and highlight the specific variable whose metadata matched the search term.

Moreover, we want to ensure that datasets hosted on the S-PHS Data Portal can be found via other discovery tools. To this end, all datasets are indexed by Google Dataset Search through the presence of well-formatted web crawler metadata, as well as by DataCite, through the issuance of DOIs and accompanying metadata. Both Google and DataCite use open and well-documented schema for their metadata, expanding the possibility for future tools to further leverage this metadata in aiding discovery.

Redivis also enables researchers to discover and explore a wide variety of data types, including geospatial, and unstructured data. Built-in previews are available for several unstructured data types (images, text, PDF, HDF5, and DICOM), and any arbitrary file type can be uploaded and stored. Redivis automatically captures and displays metadata for uploaded data, such as univariate statistics for tabular data, geospatial dispersions for geographic information system (GIS) data, and file metadata and checksums for unstructured data.

### Accessible

Access requirements are complex and often cumbersome. The Redivis system has allowed us to streamline and standardize these steps, eliminate duplicative effort for both the researcher and administrator, and ensure compliance. For tabular data, the S-PHS Data Portal automatically pulls and visualizes metadata. Metadata can also be loaded into the portal as a separate file. The system allows an administrator to set the “least necessary” requirements so that a researcher can see as much information up-front as possible to assess the suitability of the data for their research question(s).

At S-PHS’s inception, the task of tracking and verifying that data access requirements were complete and current was managed in a tabular database (REDCap) [15,16]. Although excellent for data collection, this type of software was not designed to manage data access. Because this approach was tedious, time-consuming, and often required duplicate efforts for both the data administrator and the researcher, data access was one of the first tasks S-PHS sought to automate. The system tracks which requirements an investigator has already completed so that application for an additional dataset only requires completion of new requirements specific to that dataset. Requirements for access are made transparent to investigators, and the system is designed to be user-friendly to minimize friction for investigators and administrators.

**Figure 3.**
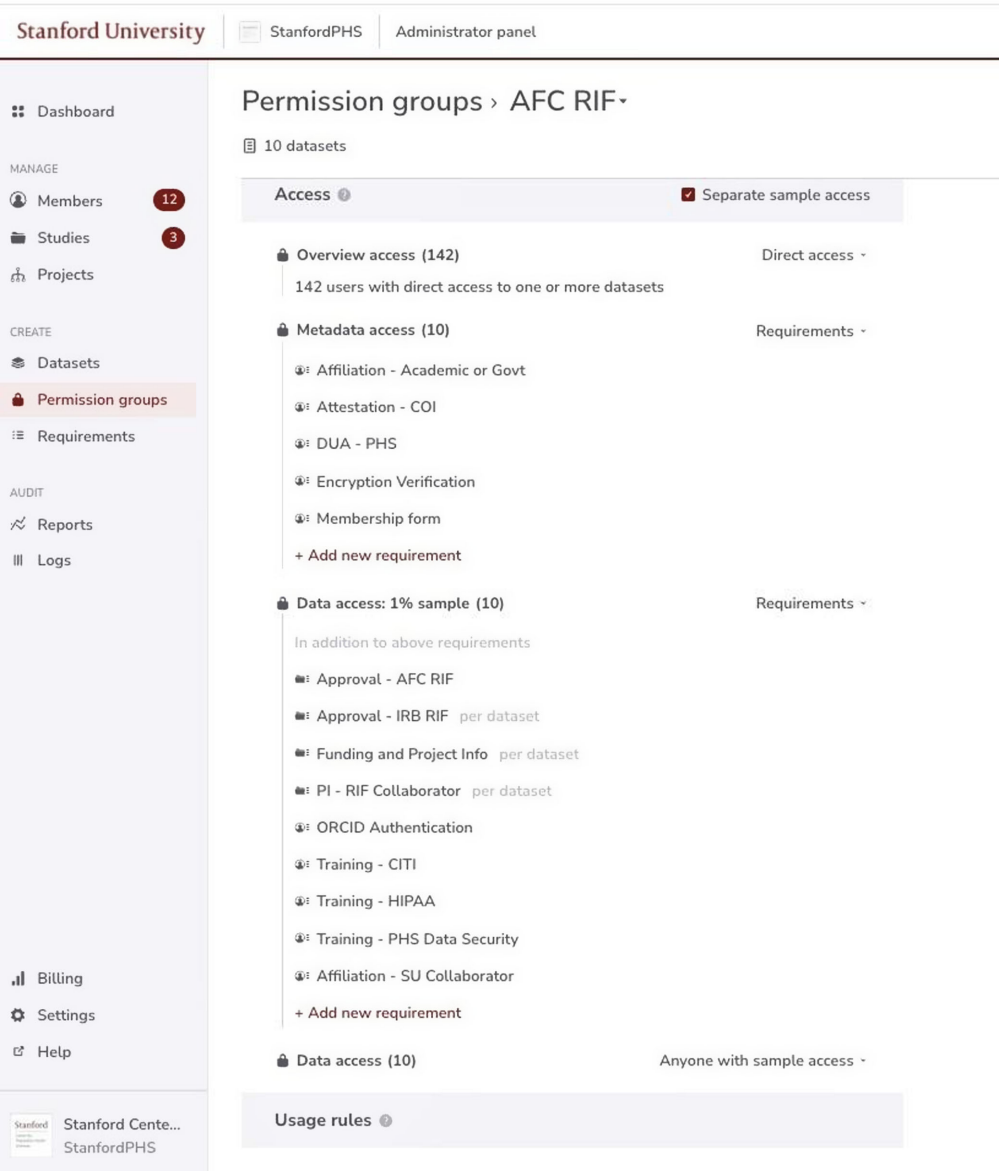
Tiered access on the S-PHS Data Portal.

Data access is managed with a customizable, tiered, granular permissioning system that allows data custodians to set specific requirements for different tiers of data visibility and access (Fig. 3). These tiers are overview, which includes detailed descriptions of data provenance and methods for collection, and other narrative information; metadata view, which includes variable descriptions and characteristics; data sample access (if, for example, the data owners have elected to make a 1% or 5% sample available prior to releasing the full dataset); and full data access. It is also possible to directly upload a dataset and share it with individual collaborators. For especially sensitive datasets, it’s possible to hide them from the search tool, and specify which individuals will be able to discover the data. All of these tiers and settings may be combined as needed to comply with security regulations or contractual requirements. It is also straightforward to make low-risk datasets publicly available.

**Figure 4.**
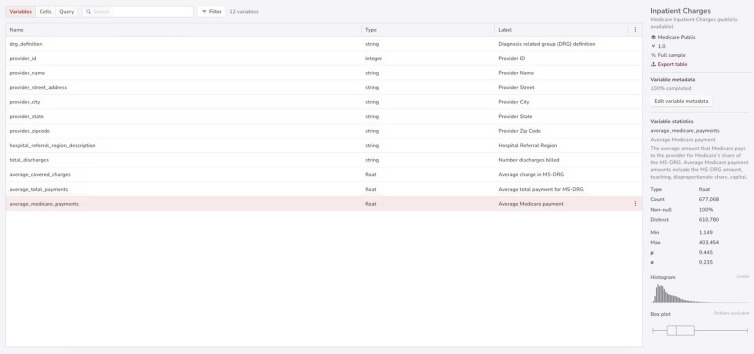
Metadata view on the S-PHS Data Portal.

The S-PHS Data Portal metadata view enables researchers to assess the utility of a dataset for their research question, and includes information such as variable names, descriptions, summary statistics, join fields, and other population-level information (Fig. 4). Searches can be performed on metadata such as tags, variable names, timeframe, dataset size, and any other information included in the metadata. Once a dataset is identified, researchers can view a more detailed description of the data provenance and methodology, a list of all variables, visualizations of key summary statistics (e.g., counts, missingness, range, mean, median, histogram, and box-plot) for each variable, and a list of the access requirements that are necessary to gain access to the dataset.

**Figure 5.**
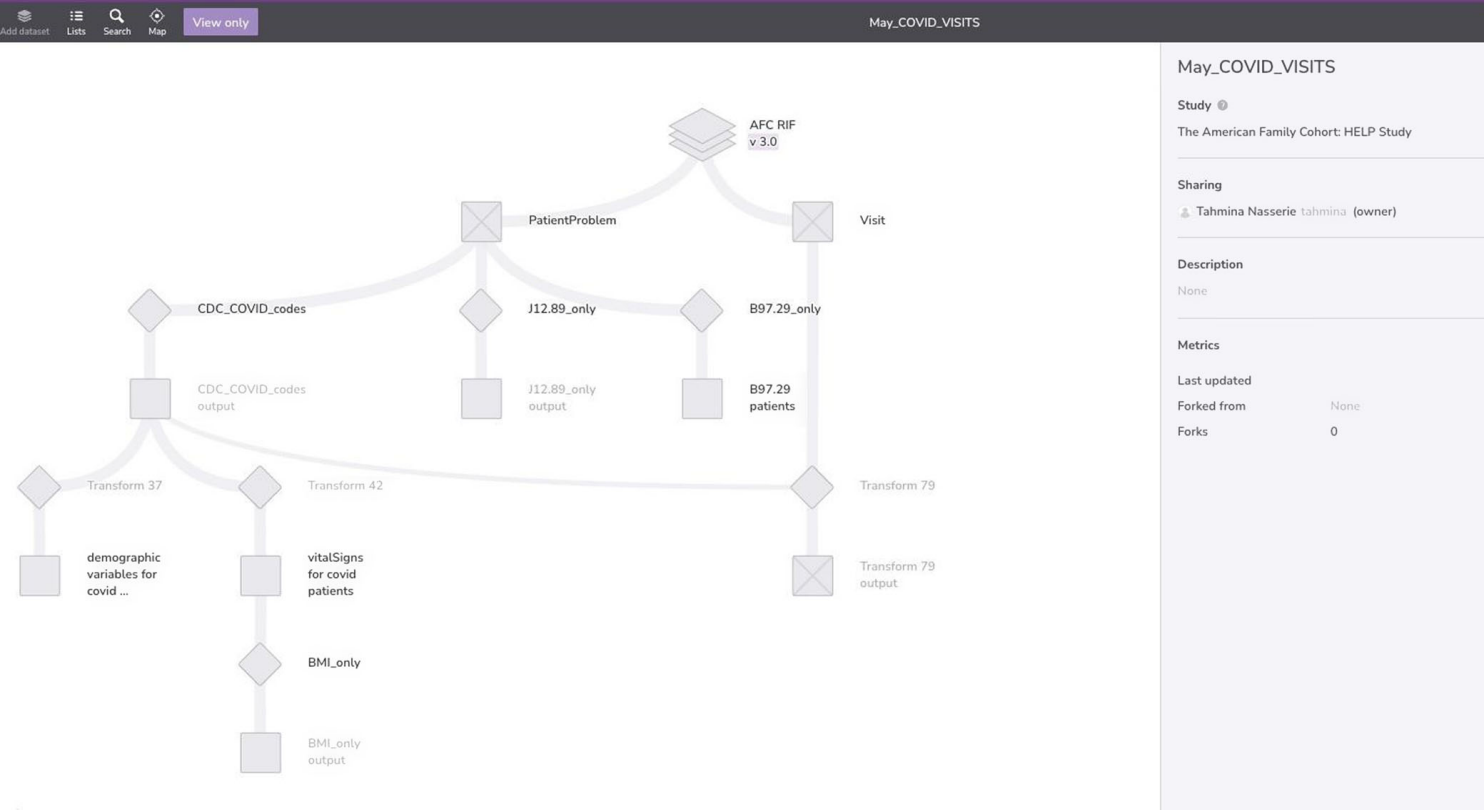
Project tool for data curation and cohort selection.

Once researchers have completed all data access requirements necessary for the 1% or 5% sample, or the full dataset, they can use graphical and SQL interface tools (Fig. 5) to identify their analytic cohort. They can select tables, variables, and records of interest, and reshape, clean, and prepare the data for analysis. Because Redivis is a software layer built on the Google Cloud Platform, all computations on both Redivis and Nero are performed through the Google Cloud Platform, and interfaced through the user’s browser [17]. All projects on Redivis can be shared with collaborators, and – similarly to collaborative work tools such as Google Docs – multiple users can communicate within a project.

Once an investigator has selected the appropriate files, variables, and records using the GUI or SQL tools within Redivis, data can be ported to any of several secure computational environments, which have a wide range of analytic tools.

### Interoperable: built for collaboration

A collaborator accessing a dataset from another institution can authenticate using their institutional ID, if they have an account and the hosting institution permits view or access of data by external collaborators. Redivis authenticates users with InCommon [18,19], which is an access and identity management service specializing in academic and government organization authentications. Because it is built on the Google Cloud Platform, it is also possible to compute on data hosted in two different institutions’ instances without moving the data, provided that all data access requirements have been met and all necessary approvals have been received. This addresses a major barrier to data sharing: institutions and investigators often do not want to let high-value data out of their direct control, and in many cases, are not permitted to do so, preventing computation by researchers from other institutions. Other organizations, such as TriNetX [20] and Google Data Commons [21], also use federated data lakes, but theirs are somewhat different from the S-PHS approach in that other platforms tend to have binary data access (all or nothing), only offer this capability for one type of data (such as EHR or claims), and are more likely to make only low to moderate-risk data available.

Prior to loading on our portal, data undergo curation for research use. A dedicated data management team at S-PHS coordinates all data transfers from data proprietors, and is responsible for removing person-readable identifiers, appending encrypted linkage keys, cleaning, curation, and all linkages that require access to explicit identifiers. This team also coordinates de-identification, conversion into common data models such as the Observational Medical Outcomes Partnership (OMOP) model, and other curation, annotation, and documentation activities in a dedicated, secure computational environment. For data managers and custodians, there are tools for data ingestion and curation, data annotation, and other data management tasks. Redivis automatically tracks contributors to datasets, and ties those contributions (including associated ORCIDs) to the DOI metadata. This means that when a paper cites a dataset, we can link back to the people who curated and contributed to the dataset. This will enable S-PHS to pull lists of all papers derived from particular datasets (any dataset that a data manager or researcher has contributed to), which could in turn provide credit to contributors of data, and career advancement related to data sharing.

### Reusable: computational environments

Once the files, variables, and records for an analytic cohort have been selected, the cohort can be moved to any of several secure computational environments depending on the needs of the research team and the data’s risk profile. The Redivis platform offers the ability for investigators to initialize Jupyter Notebooks in R, Python, and Stata. To accommodate larger data derivatives, as well as the growing prevalence of computationally intensive AI and ML methodologies, Redivis notebooks allow investigators to customize their notebook computational environments to the needs of their data and analytic methods, while billing investigators directly for these computational resources.

In addition to the Redivis native Jupyter notebooks, Stanford has three high-performance computational environments for high-risk data: a secure, on-premise cluster of Windows servers (i.e. the S-PHS Windows Servers); Carina, which is an on-premise cluster of secure Linux servers; and Nero, which is based in the Google Cloud Platform. Administrators can specify the IP addresses of permissible environments for analyses, and all computational environments are designed for the highest-risk data.

The secure computational environments present a suite of analytical tools, including (but not limited to) Jupyter, R, Python, SAS, Stata, and MATLAB, all of which are accessible through a Jupyter Lab interface and the command line for all of the platforms except for the Windows servers, in which they’re accessible using their standard program interfaces. Jupyter Lab allows researchers to switch between preferred analytic tools, annotate data, and document data curation steps in a notebook format, which can easily be shared with other investigators or subsequent data users, providing practical versatility.

Finally, we have several resources available to users, including Slack channels, a centralized ticketing system for resolving technical issues (e.g. requests for additional software packages or other system updates), and online office hours. For some of the most frequently used datasets, S-PHS also hosts research consortia for researchers to meet others working on similar data or topics, ask detailed data or methodology questions, and present proposed or current projects.

## Collaborating with partners to translate real-world data into real-world impact

S-PHS partnerships have played a central role in ensuring that RWD is translated into real-world evidence and used to reduce inequities in the development, approval, and adoption of effective medical and public health interventions—a capacity we believe is critical for centers offering access to data. An important component of reducing disparities is engaging the communities that have contributed their data, particularly communities that have historically been excluded from data sources. With all RWD we work with, it is strictly forbidden to contact patients whose information is in the data. This, however, does not mean that community perspectives and interests can’t be included in projects from their inception. To do this, S-PHS works with the Stanford Office of Community Engagement to advise study teams on how to engage communities around their topics of study. Many of our projects engage with key groups who are working to implement health-promoting policies and practices for patients and communities. In collaboration with the American Board of Family Medicine, S-PHS data are being used to track racial and ethnic differences in long-COVID. S-PHS and the San Joaquin Valley Public Health Consortium, comprised of eleven local public health jurisdictions dedicated to advancing health equity, jointly assessed the impact of COVID-19 on preventive screening rates in low-income, rural areas of California’s Central Valley. S-PHS used claims data to obtain historical information for the area, and EHR data from primary care – which are updated much more frequently than claims datasets – to conduct a timely assessment. We are also conducting community-engaged work on the effects of structural racism on health, and have a project studying the impact of cash transfers on persistent poverty. Learnings from these projects will be used to inform retrospective analyses on structural racism and persistent poverty using datasets hosted by S-PHS. These and other partnerships have enabled S-PHS to implement many of the Diversity, Inclusion, and Equity recommendations developed during the 2020 NCATS meeting [22].

## Critical further new areas needed for working with real-world data

As we look to the future, we are considering multiple new approaches for how RWD can be used to support translational science, with a focus on addressing several different barriers that we have encountered while supporting over 1000 projects over the last eight years. These include how best to harmonize across datasets with differing structures and formats, develop synthetic data, provide more numerous geographic overlays to data, increase training in the use of RWD across intuitions, and support RWD analyses with interdisciplinary research teams.

Creating data structures that allow for the harmonization of data across platforms, as well as facilitating the replication of analyses across datasets, is increasingly a focus of our work. Our current focus in this area is working to convert data to the OMOP data model, in line with the choices of many large collaborative studies [23]. This has so far resulted in great efficiencies for our work, but we have also realized that OMOP conversion is a process, not a destination. Subject matter experts using our data have continued to provide feedback on how best to capture different variables in the data, and the OMOP framework is well adapted for being updated to reflect expert opinions and changes that occur in coding in different EHR platforms.

One area of new work for us that we think is particularly promising is the development of synthetic data for our most used datasets. Synthetic data refers to fake data that is generated based on real data. The goal is to retain the properties of the original data as much as possible, such that the original and synthetic data are indistinguishable in terms of distributions of variables and relationships between variables. In the context of healthcare data, the synthetic copy of the data is not subject to the Health Insurance Portability and Accountability Act (HIPAA) or General Data Protection Regulation (GDPR) rules, as it does not include real personal information. This provides the potential to simplify the process of sharing and collaborating with RWD, while still preserving patient privacy. There are useful new approaches to creating synthetic data that capitalize on recent advancements in deep learning methods to achieve synthetic copies of complex data with high fidelity to the original data source. Because the softwares used toconstruct these datasets are computationally intensive, they must be run on a commercial cloud or similar computational environment, especially if the original data are large. We are mindful of the limitations of the underlying data, and high-fidelity synthetic data are likely to recreate these biases, as the benchmark for high-fidelity synthetic data is that they perform similarly to the original data in models. While the optimal approach is to reduce selection and measurement error during the data collection process itself, we can also implement approaches for reducing some of these sources of error in the synthetic data as well. Strategies for addressing these issues include incorporating sampling weights and other adjustments into synthetic populations, and adding guidance on addressing these limitations into data documentation.

A further area of development is to focus on datasets that have geographic information to address the fact that RWD rarely includes original data collection on social and environmental determinants of health. If some level of geographic information is included in RWD, geographic overlays can be done, which greatly expands the types of determinants of health that can be studied. Even levels of geography as large as state can be beneficial for, for example, looking at the impacts of state differences in Medicaid expansion on health outcomes. Smaller levels of geography (e.g. ZIP code or census tract) allow for the examination of neighborhood effects on health and contaminants in the physical environment. Because data on health policies, social contexts, and environmental exposures are often difficult to find, and also take time to curate, focusing on providing these measures and crosswalk files for overlay can make including these factors in translational research projects much easier for researchers. We have recently, for example, calculated several area-level deprivation measures at multiple levels of geography to capture the local social environments of patients, and have made these available to researchers on our platform. We are also working to use geographic overlay data on the demographics of small areas to reweight RWD to better reflect the underlying populations.

There are many efficiencies that can be gained by supporting cross-institution collaborations on training in the use of RWD. There are currently few established curricula on the use of RWD at academic medical centers, yet there is a growing need for this unique focus in instruction. Rather than developing coursework that is institution-based, we believe it will be important to partner to develop courses with an entirely or primarily remote structure, to allow more efficient instruction across institutions. Both asynchronous online coursework and in-person short courses that bring people together across institutions will help to build critical capacity in the skillsets needed to do translational research with RWD.

We are also increasingly focused on supporting interdisciplinary teams of staff and faculty pursuing RWD projects. Up until now, the traditional model has been that a subject matter expert would work with a statistician to analyze RWD to answer a particular research question. What we have found is that there is also a need for a team member with deep knowledge of the data itself, which often rapidly increases the speed and quality of translational research that uses RWD. In addition to these three types of expertise, we want to move towards teams that also include individuals with expertise in study design, community and patient engagement, and health equity. To successfully implement these types of multidimensional teams, it will be crucial to educate researchers about the value of each of these roles, increase the capacities of individuals with expertise in these areas, and connect researchers to an assembled team at the inception of a project.

Our experiences over the past eight years have shown us how much demand there is for access to real-world data, and that if the appropriate tools and structures are in place, successful use of that data can be achieved. While the technological solutions and management structures to support researcher, data science, and community collaborations take time to develop and implement, the long-term gains for translational science have proven to be substantial.
